# Human Helicase DDX5 is Hijacked by SARS-CoV‑2 Nsp13 Helicase to Enhance RNA Unwinding

**DOI:** 10.1021/acsomega.5c04271

**Published:** 2025-07-31

**Authors:** Giovanni Barra, Alessia Ruggiero, Valeria Napolitano, Camilla Lodola, Massimiliano Secchi, Maria Michela Pallotta, Viviana Benincasa, Francesco Leone, Giovanni Maga, Rita Berisio

**Affiliations:** † Institute of Biostructures and Bioimaging, 9327 C.N.R., Napoli I-80131, Italy; ‡ Institute of Molecular Genetics, C.N.R., Pavia I-80131, Italy

## Abstract

DEAD-box protein (DDX) 5 plays important roles in multiple aspects of cellular processes that require modulation of the RNA structure. Alongside the canonical role in RNA metabolism, numerous studies have demonstrated that DDX5 influences viral infections by directly interacting with viral proteins. However, the precise functional role of DDX5 during viral infection remains largely unclear. Here, we explore the previously undiscovered ability of DDX5 to interact and synergize with the Nsp13 helicase of SARS-CoV-2. We show that DDX5 exhibits a nanomolar binding affinity to Nsp13. Also, by dissecting DDX5 in its individual domains, we show that the Nsp13–DDX5 interaction is mediated by the RecA1 domain of DDX5. Importantly, we show that DDX5 and Nsp13 synergize in unwinding double-stranded RNA. Consistent with its ability to bind Nsp13, the RecA1 domain of DDX5 acts as a weak inhibitor of the synergic action of the two helicases in the RNA unwinding process. Modeling of the DDX5–Nsp13 complex provides a plausible explanation for the synergic action of the two helicases, in a mechanism that is likely instrumental in the early stage of infection, when the concentration of Nsp13 is still low.

## Graphical abstract

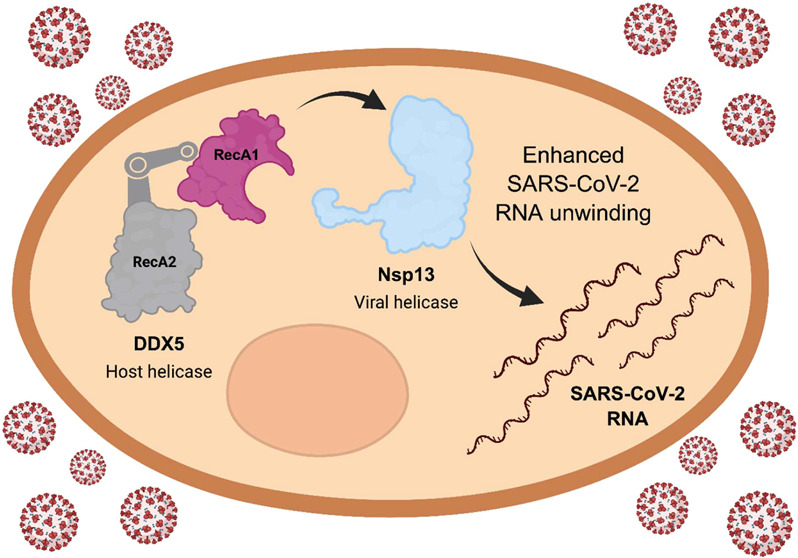

## Introduction

Human DEAD-box (DDX) helicases play an essential role in many steps of the RNA metabolism, including RNA–RNA and RNA–protein remodeling in an ATP-dependent manner.^1^ Their mutation or improper regulation is linked to pathological processes including tumorigenesis, inflammation, neurodegeneration, viral propagation, and immune response.^2–4^ DDX helicases exhibit diverse functions during viral infection, acting as either positive or negative regulators of viral replication at various stages of the viral life cycle.^5,6^ Indeed, they have been shown to differentially regulate interferon (IFN) response or other inflammatory signaling.^7^ The canonical RIG-I-like receptors (RLRs) are DDX family proteins that play pivotal roles in the cytoplasmic sensing of viral RNA, thereby triggering the expression of antiviral genes.^8^

Several RNA viruses hijack human helicases to facilitate various stages of their replication cycles. However, the specific mechanisms of these interactions and how host–pathogen dynamics influence the functional states of DDX helicases remain poorly understood.^9,10^ Notably, DDX proteins are essential for the replication of different RNA viruses, including HIV-1, HCV, Influenza A, Dengue, Infectious Bronchitis Virus (IBV)-CoV, SARS-CoV and SARS-CoV-2.^11–16^ In the case of SARS-CoV-2, several human RNA helicases were found to interact with the nucleocapsid (N) protein, such as DDX1,^13^ DHX9, DHX30 ^13^ and DDX21.^12^ Functional studies, including loss-, gain-, and reconstitution-of-function experiments, have showed that DDX1 acts as a potent anti-SARS-CoV-2 host factor by inhibiting viral replication and protein synthesis. Interestingly, the N-targeting and anti-SARS-CoV-2 abilities of DDX1 are independent of its ATPase and helicase functions. In line with these findings, DDX1 and its paralog DDX3X were shown to physically associate with nucleocapsid protein Np and to enhance its binding affinity for double-stranded RNA by two- to four-fold, through a mechanism that does not require helicase activity.^17^ Among DDX helicases, DDX5 functions as both a viral infection helper and an inhibitor, depending on the virus type. Indeed, some viruses require active DDX5 for efficient viral replication, including HIV-1, HCV,^16^ SARS-CoV,^14^ SARS-CoV-2,^11–18^ AJEV, PRRSV, and IVA. Other viruses, like HBV and myxoma virus (MYXV), require inhibition of DDX5 activity. However, the knowledge of the underlying mechanisms of DDX5 pro-viral activity during the time course of infection remains limited.

Helicases are also encoded by (+) RNA viruses with genomes larger than 7 kb. Similar to host DDX enzymes, these viral helicases are nucleic-acid-dependent ATPases capable of unwinding DNA or RNA duplexes during nucleic acid replication, transcription, DNA repair, RNA maturation, and splicing. The helicase Nsp13 of SARS-CoV-2 is part of the SF1 superfamily and can exert multiple enzymatic activities.^19^ It is able to unwind both RNA and DNA duplexes in the 5′ to 3′ direction and displays RNA 5′-triphosphatase activity, thus playing an important role in mRNA capping.^20,21^ The SARS-CoV-2 Nsp13 helicase comprises five distinct domains, organized in a triangular pyramid-like architecture.^22^ Positioned at the apex are the N-terminal zinc-binding (ZBD) and the stalk (S) domains, followed by a 1B β-barrel domain and two RecA domains, which contain the conserved residues essential for nucleotide binding and hydrolysis.^22^

Through two-hybrid assays in mammalian cells and coimmunoprecipitation experiments, DDX5 derived from A549 cells was shown to directly and specifically bind to the SARS-CoV Nsp13 helicase protein.^14^ As for SARS-CoV-2, a breakthrough in research was the determination of its interactome using affinity-purification mass spectrometry, which identified 332 high-confidence protein–protein interactions between SARS-CoV-2 and human proteins.^12^ Following studies enhanced the number of host factors interacting with SARS-CoV-2 proteins, indicating a strong role of host–pathogen interactions in the establishment of the viral infection.^23^ It is puzzling that despite the observed interaction between DDX5 and Nsp13 helicases of SARS-CoV^14^ and the high sequence identity between Nsp13 helicases of SARS-CoV and SARS-CoV-2 (99.8%), an interaction between Nsp13 of SARS-CoV-2 and DDX5 has never been reported. On the other hand, DDX5 was found to play a pro-viral role by facilitating SARS-CoV-2 infection.^11–18^ Also, viruses have an intimate need for RNA helicases, and a synergic interaction between viral and host helicases may be insightful, as it could be instrumental to regulate the unwinding of the large viral genome, especially in the early stage of infection, when the level of viral helicases is poor. To attempt an answer to the possible role of the DDX5 helicase in providing a hijacking vehicle to Nsp13 for SARS-CoV-2 replication enhancement, we biophysically and biochemically studied the interaction of recombinant Nsp13 with more variants of human DDX5 with different complexity. Our results point to an important role of DDX5 helicases in enhancing the Nsp13 RNA unwinding activity through its direct high affinity interaction.

## Results

### Human RNA Helicase DDX5 is a Stable Multidomain Enzyme

DDX5 is a 614-residue protein with a modular arrangement. Sequence analysis using the PFAM database allows the identification of two well-defined RecA domains followed by a long, nonstructured C-terminal end (Figure 1A). Experimental structural information is available only for the domain RecA1 and part of the variable N-terminal region of DDX5.^24^ Therefore, we used artificial intelligence (AI) and the program AlphaFold3.0 to model the DDX5 structure in complex with ATP.^16^. The resulting structure was highly reliable (plDDT in the range 70–100), for the protein region 38–478, whereas the remaining regions displayed elevated errors (plDDT < 50) (Figure S1), consistent with the secondary structure predictions, using JPRED.^25^ The ATP molecule binds to the RecA1 domain through several hydrogen bonds. The adenine base of ATP forms hydrogen bonds with the main chain of Glu116 and the side chain of Gln121 and a stacking interaction with the side chain of Phe114. Phosphates 1 and 2 form hydrogen bonds with Ser142, Lys144, and Thr145 (Figure 1B). To dissect the importance of individual domains in the protein stability and in their possible role in hijacking the Nsp13 helicase of SARS-CoV-2, we recombinantly produced DDX5 and smaller variants at lower molecular complexity (Figure 1A), namely, a full-length protein (flDDX5), a variant depleted of the flexible C-terminal end (DDX5_ΔC_), and individual RecA1 and RecA2 domains (Figure 1A). Far-UV CD spectroscopy spectra have proven that expressed and purified DDX5 variants have well-structured folds with high α-helical content, with typical minima at 208 and 222 nm (Figure 2). CD spectra show that the removal of the C-terminal arm in DDX5Δ*C* does not affect the protein stability (Figure 2A,B). Consistently, thermal unfolding curves recorded for both proteins by following the CD signal at 222 nm as a function of temperature exhibit the characteristic sigmoidal profile expected of two-state systems, with the same melting temperature *T*_m_ = 52 °C (Figure 2C,D). Also, dissection of the RecA1 and RecA2 domains of DDX5 does not negatively impact protein stability. Indeed, *T*_m_ values computed for the thermal denaturation curves of RecA1 and RecA2 are 55 and 58 °C, respectively (Figure 2G,H).

**Figure 1:**
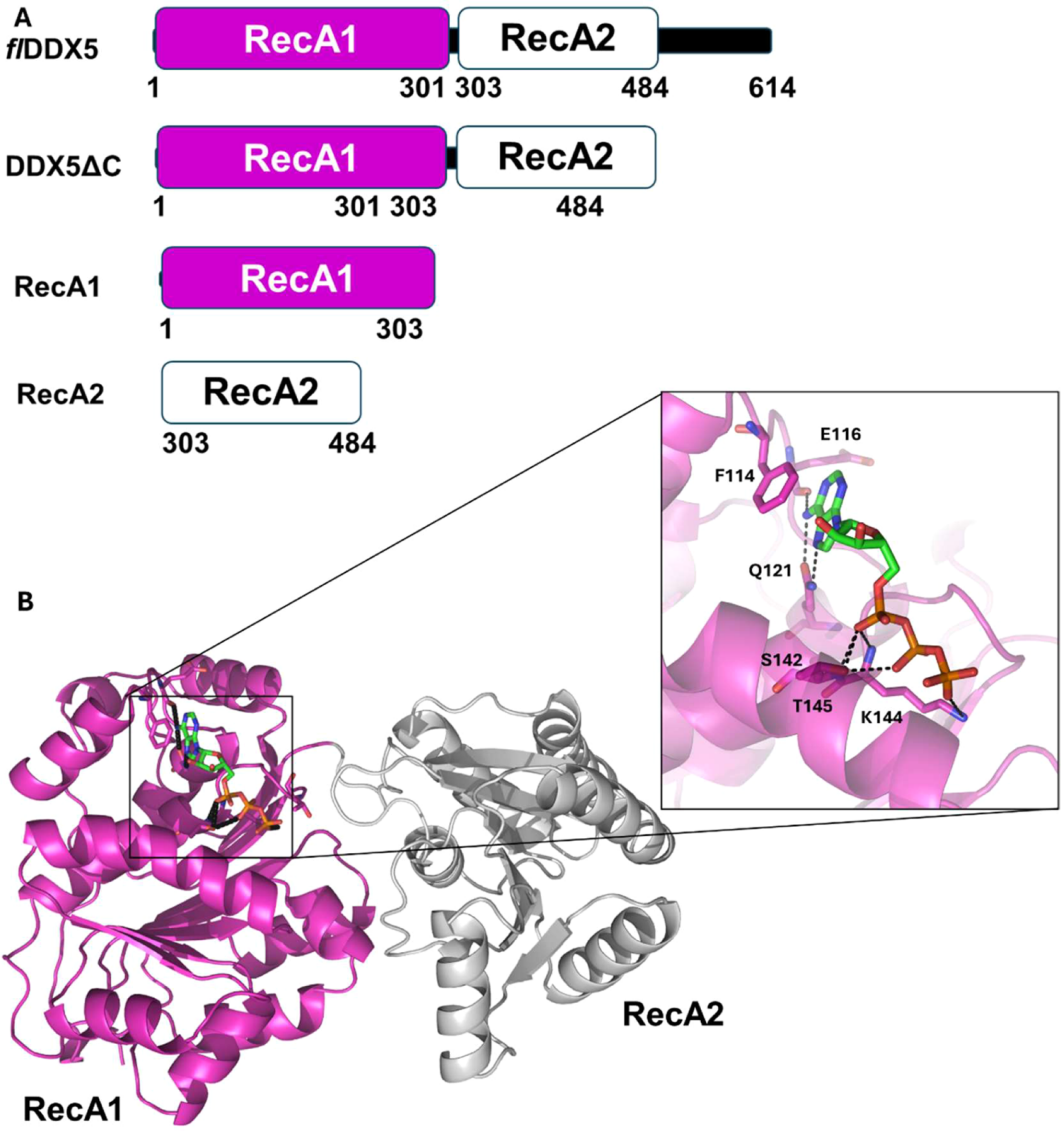
Domain architecture of the DDX5 helicase. (A) DDX5 variants produced in this study. (B) Cartoon representation of the DDX5 structure model in complex with ATP, generated using AlphaFold3.0. The ATP molecule and its contacting residues are drawn in stick representation; a zoomed-in view is reported in the inset.

**Figure 2:**
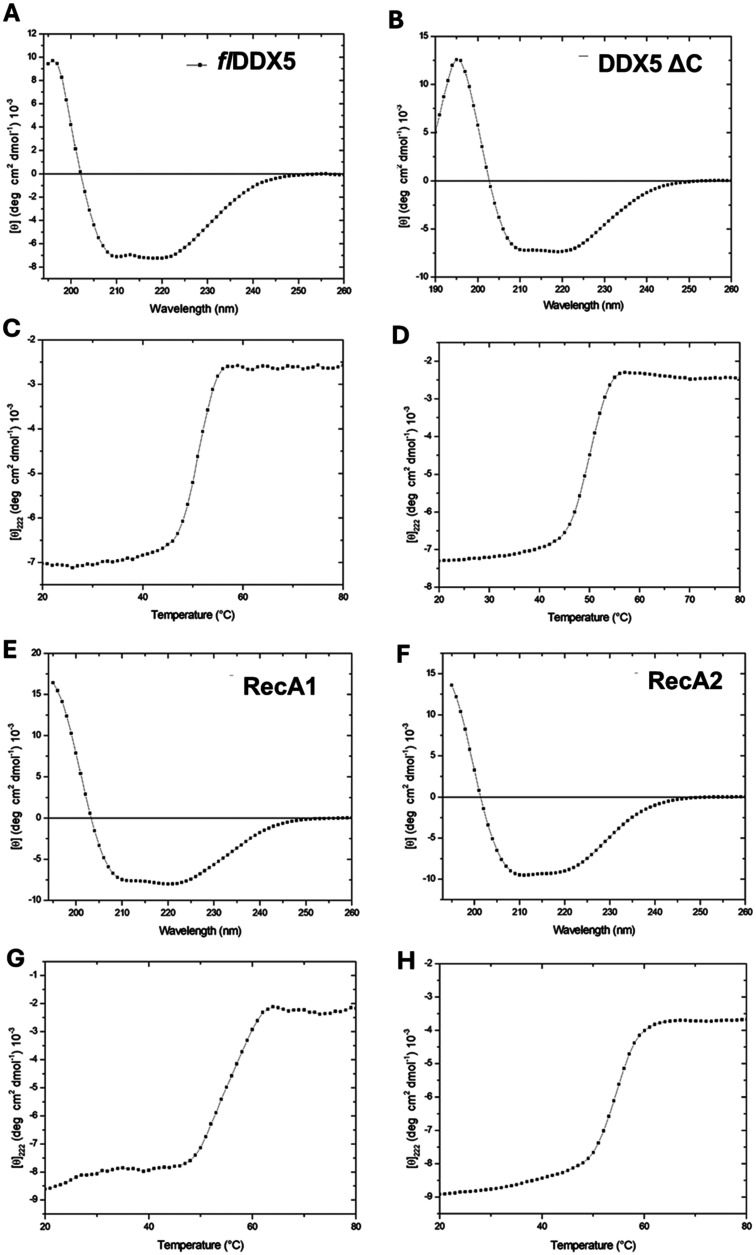
CD spectroscopy studies of DDX5 and its variants. CD spectra recorded at 20 °C for full-length DDX5 (1–614) (A), its C-terminally truncated form DDX5Δ*C*(1–484) (B), RecA1 domain (1–303) (E), and RecA2 domain (303–484) (F). The corresponding thermal denaturation curves, measured at 222 nm, are reported in panels C, D, G, and H.

### Nsp13 Binds DDX5 with Nanomolar Binding Affinity

Biolayer Interferometry (BLI) studies were performed to study the differential abilities of the different DDX5 variants to bind Nsp13. The freshly purified Nsp13 protein was covalently immobilized on an AR2G biosensor (Sartorius). Then, the kinetics of association and dissociation to the immobilized Nsp13 were measured at increasing concentrations of flDDX5, in the range between 18.7 nM and 600 nM. As shown in Figure 3A, flDDX5 bound Nsp13 in a dose-dependent manner, and data fitting obtained applying a heterogeneous 2:1 model provided a *K*_D_ of (8.1 ± 0.15)­10^–9^ M and K_D2_ of (1.2 ± 0.02) 10^–7^ M (Table 1). A nearly identical binding *K*_D_ value was measured for DDX5Δ*C* (Figure S2A), showing that the C-terminal arm (residues 485–614) has no impact on Nsp13–DDX5 interaction. The same binding experiments were conducted for the two domains, RecA1 and RecA2. Sensorgrams showed a clear dose–response binding of RecA1 to Nsp13 (Figure S2B), whereas no significant variation of sensorgrams was observed for RecA2, showing that the RecA2 domain is unable to bind Nsp13 (Figure S2C). However, data were not of sufficient quality to determine binding constants for RecA1, due to aggregation processes at high RecA1 concentrations. Therefore, we performed further binding assays using microscale thermophoresis (MST), which allows for quantitative, immobilization-free analysis of protein interactions, using low protein concentrations.

**Figure 3:**
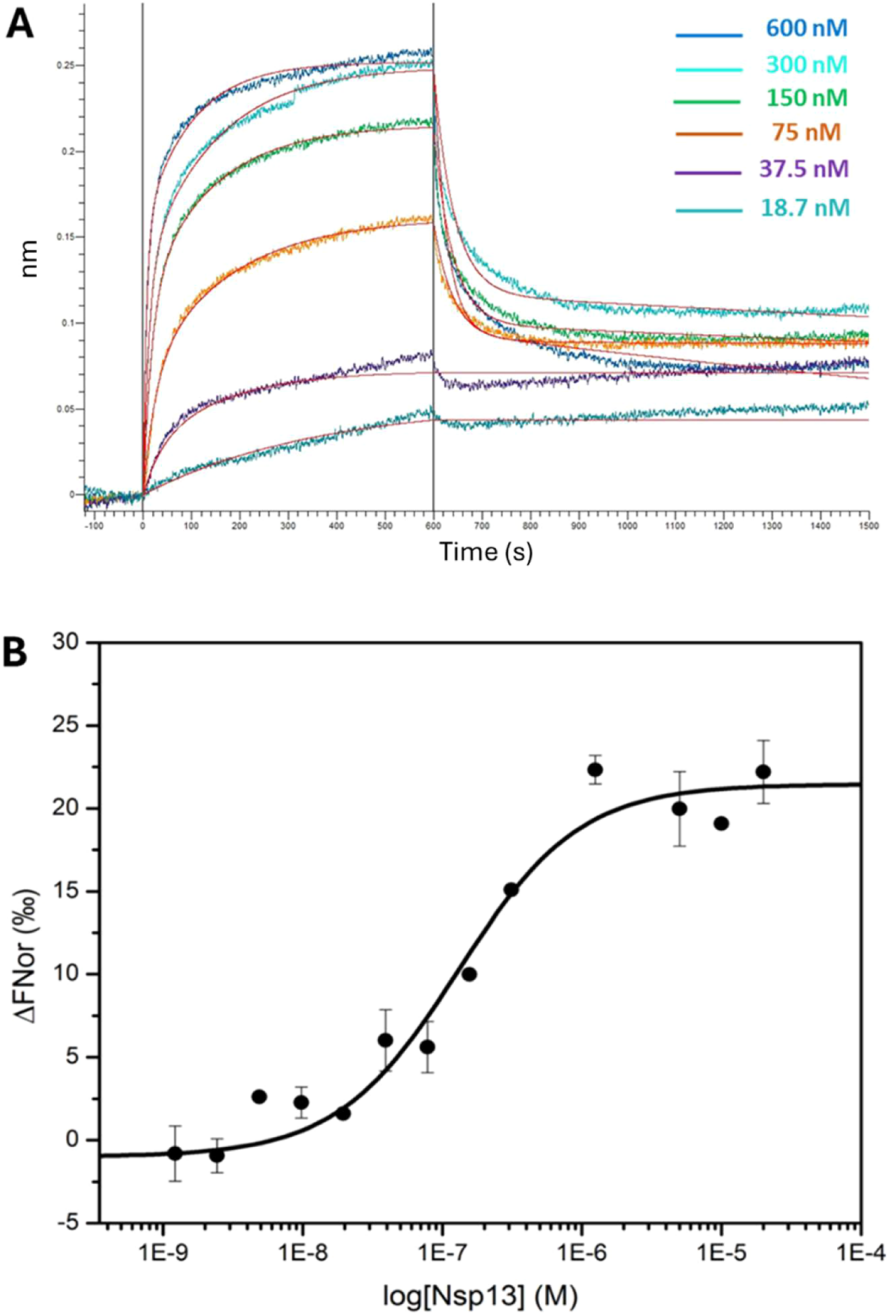
Binding between Nsp13 and DDX5 helicases. (A) Overlay of biolayer interferometry (BLI) sensorgrams obtained for the binding of different concentrations of flDDX5 to the Nsp13 immobilized on AR2G sensor chips. (B) Direct binding of Nsp13 to fluorescent flDDX5 by MST. The error bars represent the standard deviations (SD) of each data point calculated from three independent thermophoresis measurements.

**Table 1:** BLI Kinetics Parameters Related to Binding Curve Analysis between flDDX5 and Nsp13

flDDX5														
concn (nM)	*K*_D_ (M)	*K*_D2_	*K*_D_ error	*K*_D2_ error	*k*_a_ (1/Ms)	*k*_a2_	*k*_a_ error	*k*_a2_ error	*k*_dis_ (1/s)	*k*_dis2_	*k*_dis_ error	*k*_dis2_ error	full *X* ^2^	full *R* ^2^
18.7					1.526 × 10^5^	1.401 × 10^5^							01205	08986
37.5	<1.0 × 10^–12^	1.222 × 10^–11^	<1.0 × 10^–12^	1.222 × 10^–11^	1.705 × 10^5^	7.572 × 10^5^	7.723 × 10^3^	7.312 × 10^4^	<1.0 × 10^–7^	9.254 × 10^–6^	<1.0 × 10^–7^	9.211 × 10^–7^	0.1545	08605
75	2.029 × 10^–10^	1.157 × 10^–7^	2.545 × 10^–11^	2.133 × 10^–9^	7.540 × 10^4^	2.098 × 10^5^	3.600 × 10^2^	3.669 × 10^3^	1.530 × 10^–5^	2.426 × 10^–2^	1.918 × 10^–6^	1.417 × 10^–4^	0.0383	0.994
150	2.684 × 10^–9^	1.275 × 10^–7^	6.052 × 10^–11^	1.834 × 10^–9^	4.723 × 10^4^	2.032 × 10^5^	2.803 × 10^2^	2.693 × 10^3^	1.268 × 10^–4^	2.591 × 10^–2^	2.758 × 10^–6^	1.450 × 10^–4^	0.0922	0.9953
300	6.065 × 10^–9^	1.233 × 10^–7^	1.408 × 10^–10^	1.806 × 10^–9^	2.241 × 10^4^	1.963 × 10^5^	1.486 × 10^2^	2.611 × 10^3^	1.359 × 10^–4^	2.420 × 10^–2^	3.024 × 10^–6^	1.486 × 10^–4^	0.151	0.994
600	2.351 × 10^–8^	2.195 × 10^–7^	3.703 × 10^–10^	4.225 × 10^–9^	1.636 × 10^4^	1.738 × 10^5^	1.543 × 10^2^	3.074 × 10^3^	3.846 × 10^–4^	3.814 × 10^–2^	4.852 × 10^–6^	2.896 × 10^–4^	0.2488	0.9945
**Av**.	**8.115** × **10** ^**–9**^	**1.172** × **10** ^**–7**^	**1.493** × **10** ^**–10**^	**2.002** × **10** ^**–9**^	**8.076** × **10** ^**4**^	**2.801** × **10** ^**5**^	**1.773** × **10** ^**3**^	**1.703** × **10** ^**4**^	**1.656** × **10** ^**–4**^	**2.251** × **10** ^**–2**^	**3.138** × **10** ^**–6**^	**1.452** × **10** ^**–4**^	**0.1342**	**0.9561**

For the MST experiment, the concentration of fluorescently labeled flDDX5 was kept constant (5 nM), while Nsp13 concentration varied from 0 to 20 μM. The results show that increasing amounts of Nsp13 clearly affect the thermophoretic motion of DDX5. Binding of Nsp13 to DDX5 yielded a *K*_D_ value of 129.9 ± 26.0 nM (Figure 3B).

In the next step, a competitive MST method was applied to determine the binding affinity of RecA1 and RecA2 domains. The principle of this assay is that the formation of a new complex between Nsp13 and the analytes displaces fluorescent flDDX5, resulting in an altered fluorescence signal. To establish this assay, we determined, from the previous experiment, the concentration of Nsp13 giving 80% saturation (800 nM) and used fluorescently labeled flDDx5 as the tracer (Figure 3B). Therefore, 800 nM of Nsp13 was mixed with 5 nM of the tracer, and then, serial dilutions of unlabeled flDDX5 (3–0 μM), RecA1 (20–0 μM), and RecA2 (55–0 μM) were added in parallel experiments. The first experiment using unlabeled flDDX5 confirmed that unlabeled flDDX5 is able to displace labeled flDDX5 with a nanomolar IC50 value of 325.3 ± 38.5 nM (Figure 4A). To control that the signal change is not due to the self-association of unlabeled flDDX5 to fluorescently labeled flDDX5, we made sure that the MST signal decreases upon addition of unlabeled DDX5 but not upon addition of Nsp13 (data not shown). As shown in Figure 4B, the addition of RecA1 is able to disrupt the DDX5–Nsp13 complex in the competitive MST analysis, with IC50 = 16.2 ± 8.8 μM. On the other hand, even elevated concentrations (up to 55 μM) of RecA2 had no significant effect (Figure 4C).

**Figure 4:**
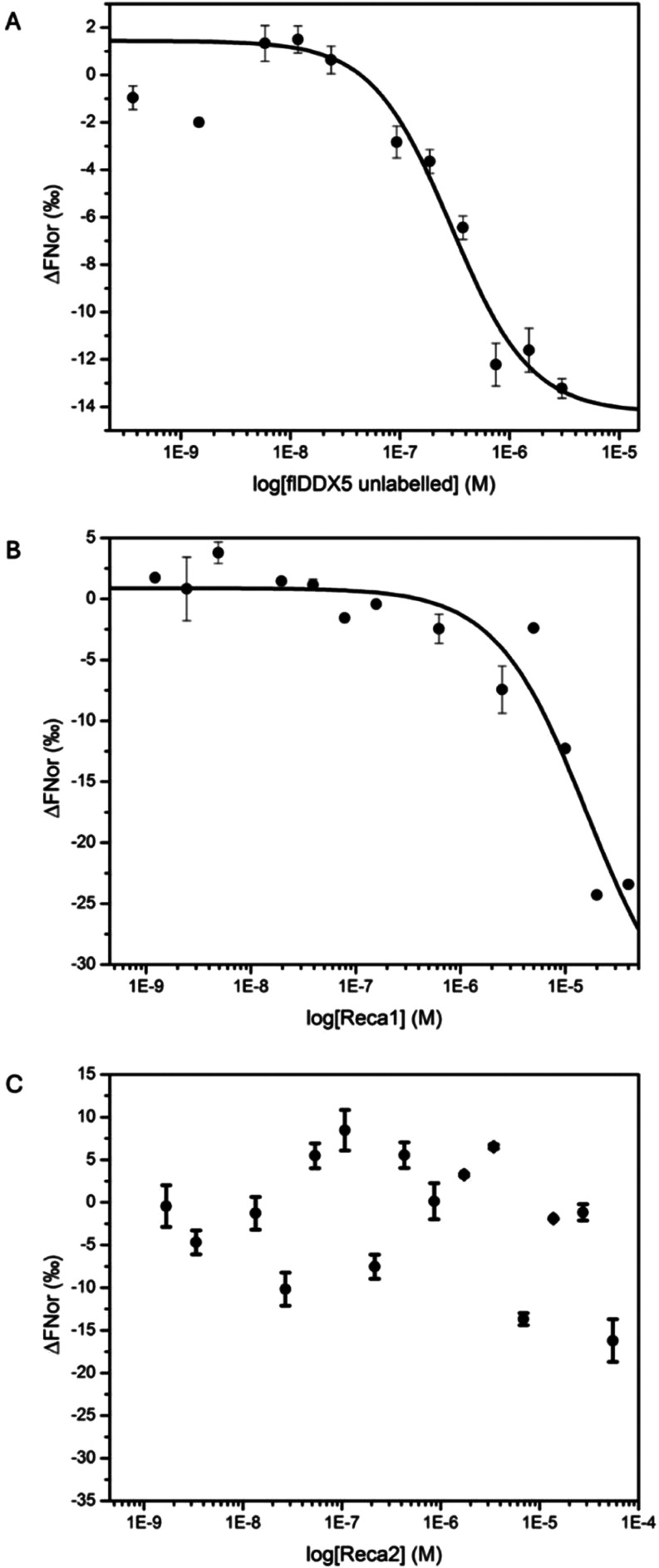
Displacement of flDDX5 in a microscale thermophoresis assay. Data are given as the mean ± SD of triplicates. Displacement (A) by flDDX5, IC50 = 325.3 ± 38.5 nM and (B) by the RecA1 domain, IC50 = 16.2 ± 8.8 μM. No displacement by the RecA2 domain was observed (C).

To further validate our findings, we examined the direct binding between labeled RecA1 and Nsp13 using MST (Figure S3). The dissociation constant (*K*_D_ = 9.1 ± 1.3 μM) closely aligns with the IC50 value obtained from the displacement assay, supporting the reliability of our measurements. These data, showing the binding of RecA1 and not of RecA2 to Nsp13, are in accordance with the BLI results (Figure 3A). No aggregation was observed in any run, even at concentrations required to reach a plateau.

### Nsp13 and DDX5 Synergize in Unwinding RNA

We aimed to investigate whether binding of DDX5 to Nsp13 enhances its RNA helix unwinding activity. An 18/38 mer RNA (dsRNA) helix substrate was used to assess helicase unwinding efficiency of RNA helicases, as reported previously.^17^ In parallel assays, the RNA substrate was incubated with either the Nsp13, DDX5, or Nsp13–DDX5 complex while maintaining a constant total helicase concentration. As shown in Figure 6, Nsp13 and DDX5 0.1 μM showed a comparable RNA unwinding activity, of 55% (Figure 5, lanes 2,3). The incubation of RNA with a mixture of 0.05 μM Nsp13 and 0.05 μM DDX5 produced an increase in the percentage RNA unwinding activity to 75% (Figure 5, lanes 4). The same synergistic action of Nsp13 and DDX5 was observed when a total helicase concentration of 0.2 μM was used (Figure 5, lanes 5–7).

**Figure 5:**
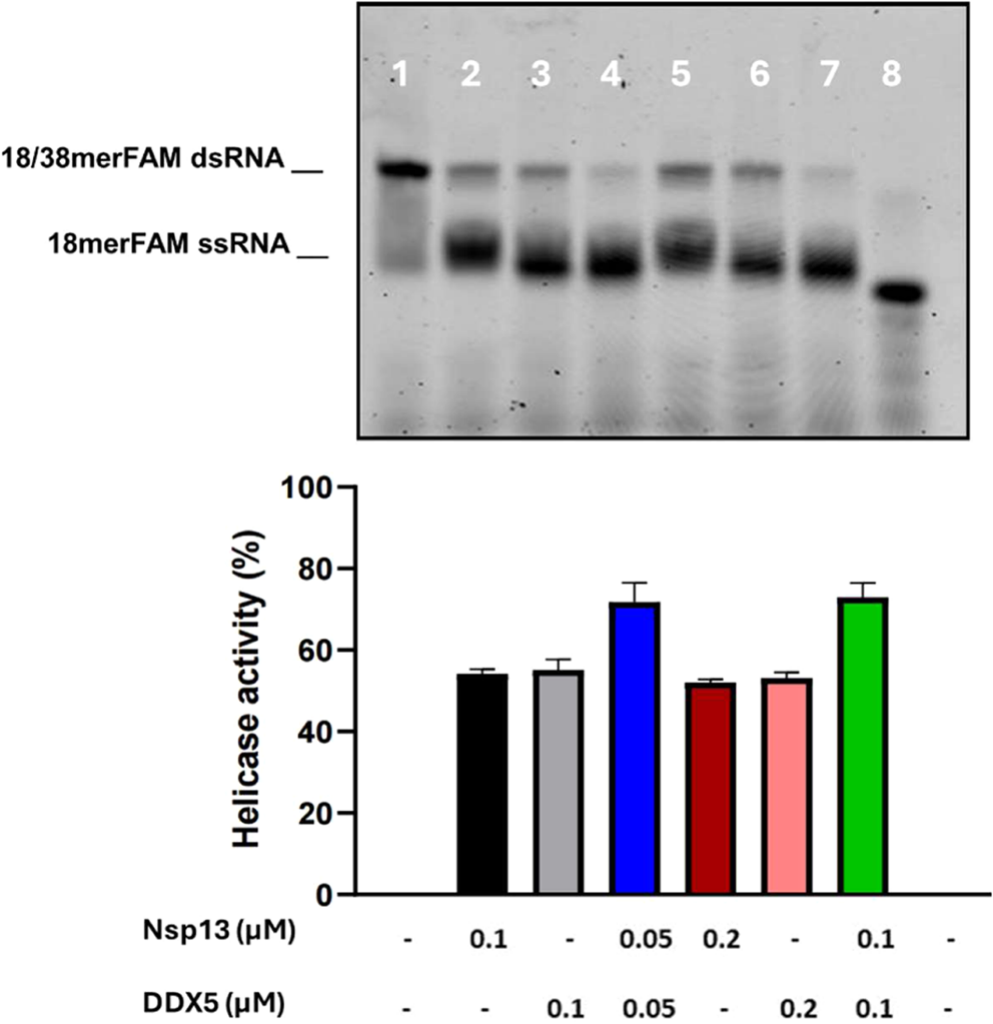
Synergic effect of Nsp13 and DDX5 interaction on the helicase activity. Top panel: Native PAGE analysis of the helicase reaction in the presence of Nsp13 (lanes 2 and 5), DDX5 alone (lanes 3 and 6), or a combination of both (lanes 4 and 7). The controls 18/38 mer dsRNA and 18 mer ssRNA in the absence of proteins are in lanes 1 and 8, respectively. Bottom panel: Percentage of helicase activity. Data points represent the average of three independent experiments ± SD; graphic bars were drawn by using the software GraphPad Prism 9.0.

We then evaluated whether the two isolated domains, RecA1 and RecA2, affected the helicase activity of the Nsp13–DDX5 complex. As expected, neither the isolated RecA1 nor the RecA2 domains are endowed with significant RNA unwinding activity (Figure 6A,B, lane 4). However, the addition of RecA1 from 0.1 to 0.5 μM depresses the unwinding activity of Nsp13–DDX5 from 85% to 70% (Figure 6A, lanes 5–8). This result suggests that the binding of the RecA1 domain to Nsp13 partially displaces DDX5 from the Nsp13–DDX5 complex, thus reducing the observed synergy of the two enzymes. Differently, the addition of the RecA2 domain has no effect on Nsp13–DDX5 helicase activity (Figure 6B, lanes 5–8), in line with its inability to bind Nsp13 helicases (Figure S2).

**Figure 6:**
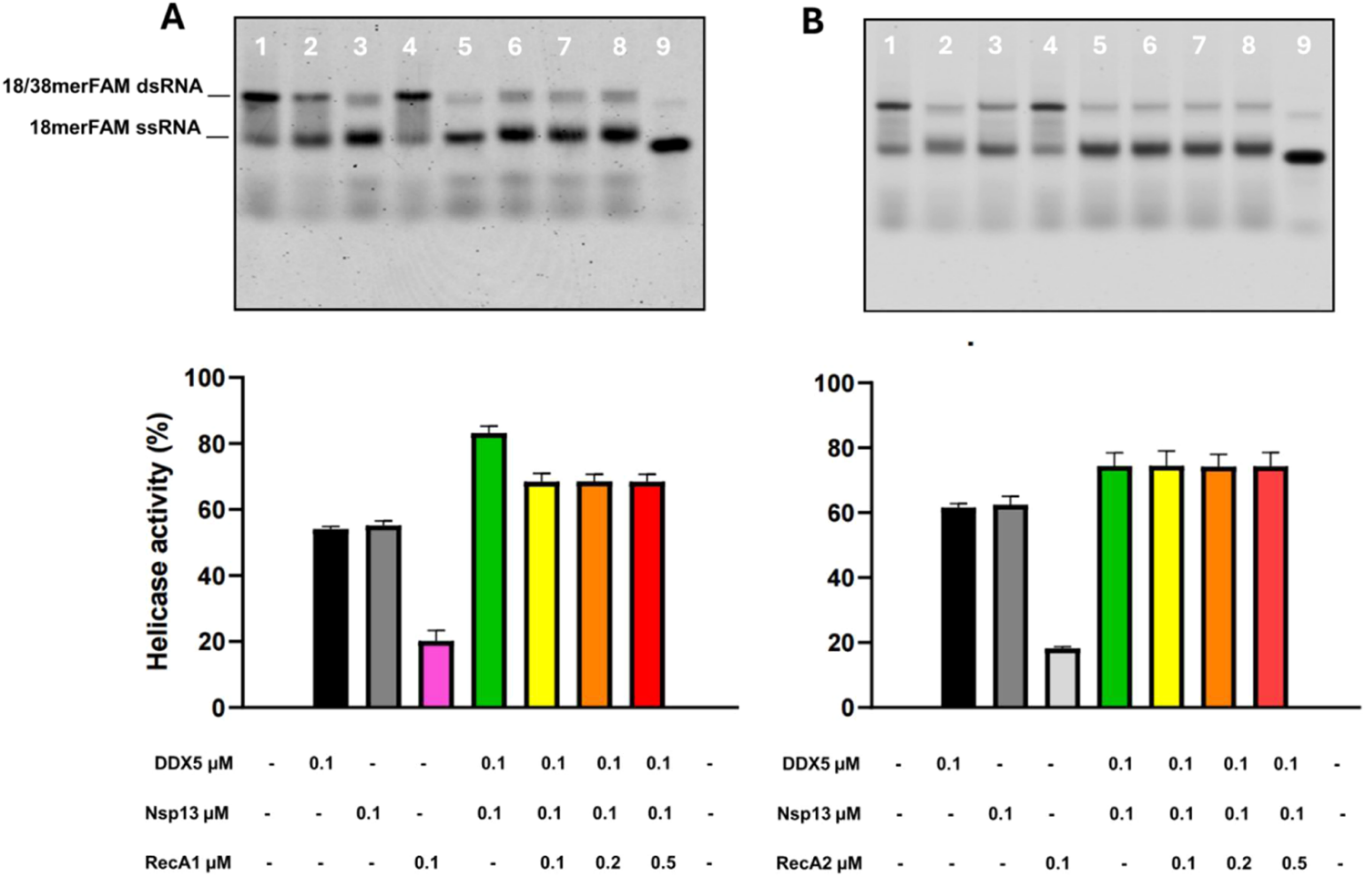
Effect of RecA1 and RecA2 domains on the helicase activity of the Nsp13–DDX5 complex. (A) Top: Native PAGE analysis of the helicase reaction in the presence of Nsp13 (lane 2), DDX5 alone (lane 3), RecA1 alone (lane 4), Nsp13–DDX5 complex alone (lane 5), and RecA1 from 0.1 to 0.5 μM (lanes 6–8). Bottom: Percentage of helicase activity. (B) Top: Native PAGE analysis of the helicase reaction in the presence of Nsp13 (lane 2), DDX5 alone (lane 3), RecA2 alone (lane 4), Nsp13–DDX5 complex alone (lane 5), and RecA2 from 0.1 to 0.5 μM (lanes 6–8). Bottom: Percentage of the helicase activity. Data points represent the average of three independent experiments ± SD; graphic bars were drawn using the software GraphPad Prism 9.0.

## Discussion

The human RNA helicase DDX5 promotes viral infection via regulating N6-methyladenosine levels on the DHX58 and NFκB transcripts to dampen antiviral innate immunity.^26^ Its homologous helicase in SARS-CoV-2 was shown to hijack the host through direct interactions with EWSR1 (Ewing Sarcoma breakpoint region 1/EWS RNA binding protein 1)^27^ and with the deubiquitinase USP13;^28^ both interactions promoting viral replication. Here, we uncovered a previously uncharacterized interaction between Nsp13 of SARS-CoV-2 and the host DDX5 helicase. This interaction, which was determined by interferometric (BLI) and thermophoresis (MST) approaches, is characterized by a strong binding affinity with a dissociation constant *K*_D_ in the nanomolar range. By dissecting the DDX5 sequence in its isolated domains, we proved that binding mainly occurs through the N-terminal RecA1 domain of DDX5. Also, using a biochemical RNA unwinding assay, we show that DDX5 and Nsp13 synergically unwind dsRNA.

Prior studies established that two copies of Nsp13 helicases form a stable complex with the replication transcription complex (RTC), which includes one copy of Nsp7, two copies of Nsp8, and one copy of Nsp12. In this Nsp13_2_–RTC complex, only one copy engaged in RNA binding.^29^ In the RNA-engaged Nsp13, the dsRNA is enclosed in a tunnel between the two RecA domains and the 1B domain.^29^ To investigate the structural determinants of the interaction and synergy between the two helicases, we modeled a full complex including DDX5, Nsp13, a double-stranded RNA, and ATP, using AlphaFold3.0 (Figure 7).^30^ Consistent with our BLI and MST binding analysis, modeling studies suggest that the direct DDX5 interaction with Nsp13 is mediated solely by its RecA1 domain (Figure 7). Although this model is purely speculative, it is fully consistent with the current knowledge of the Nsp13 residues involved in RNA binding, mainly including residues from the catalytic RecA1 and RecA2 domains and the 1B domain (Figures 7 and S4).^22–24,26,27,27–29^ Consistent with cryo-EM studies,^29^ the 1B domain of Nsp13 plays an important role in closing the RNA binding groove of the RNA-engaged Nsp13 (Figure 7). On the other hand, Nsp13 interactions with the RecA1 domain of DDX5 mainly occur through its N-terminal zinc-binding domain (ZBD) (Figure 7). Interestingly, in the Nsp13_2_–RTC complex structure, two protomers of Nsp13 sit on top of the RTC, with each ZBD interacting with one of the two N-terminal helical extensions of Nsp8 [20]. This points to the ZBD domain of Nsp13 as a possible regulator of transcription in SARS-CoV-2 through interactions with the RTC or other viral or host proteins. Consistently, ZBD domains are known to act as an anchor domain for the binding of different transcription factors during the regulation of transcription initiation, recycling, and termination processes.^31^ In the DDX5–Nsp13 complex, anchoring through the ZBD domain allows the two helicases to attach the RNA double helix in two adjacent sites (Figure 7), in a binding fashion likely responsible for the enhancement of RNA unwinding activity. However, future studies are needed to establish whether DDX5 acts as a shuttle of Nsp13 to the RTC, through its concurrent interactions with dsRNA (Figure 7), or if it plays an active helicase role in the context of the RTC machinery. Finally, the data presented here suggest an important role of host hijacking by Nsp13 in the pro-viral action of DDX5 during SARS-CoV-2 infection,^14^ through the exploitation by SARS-CoV-2 of the host’s ability to unwind RNA for its own replication.

**Figure 7:**
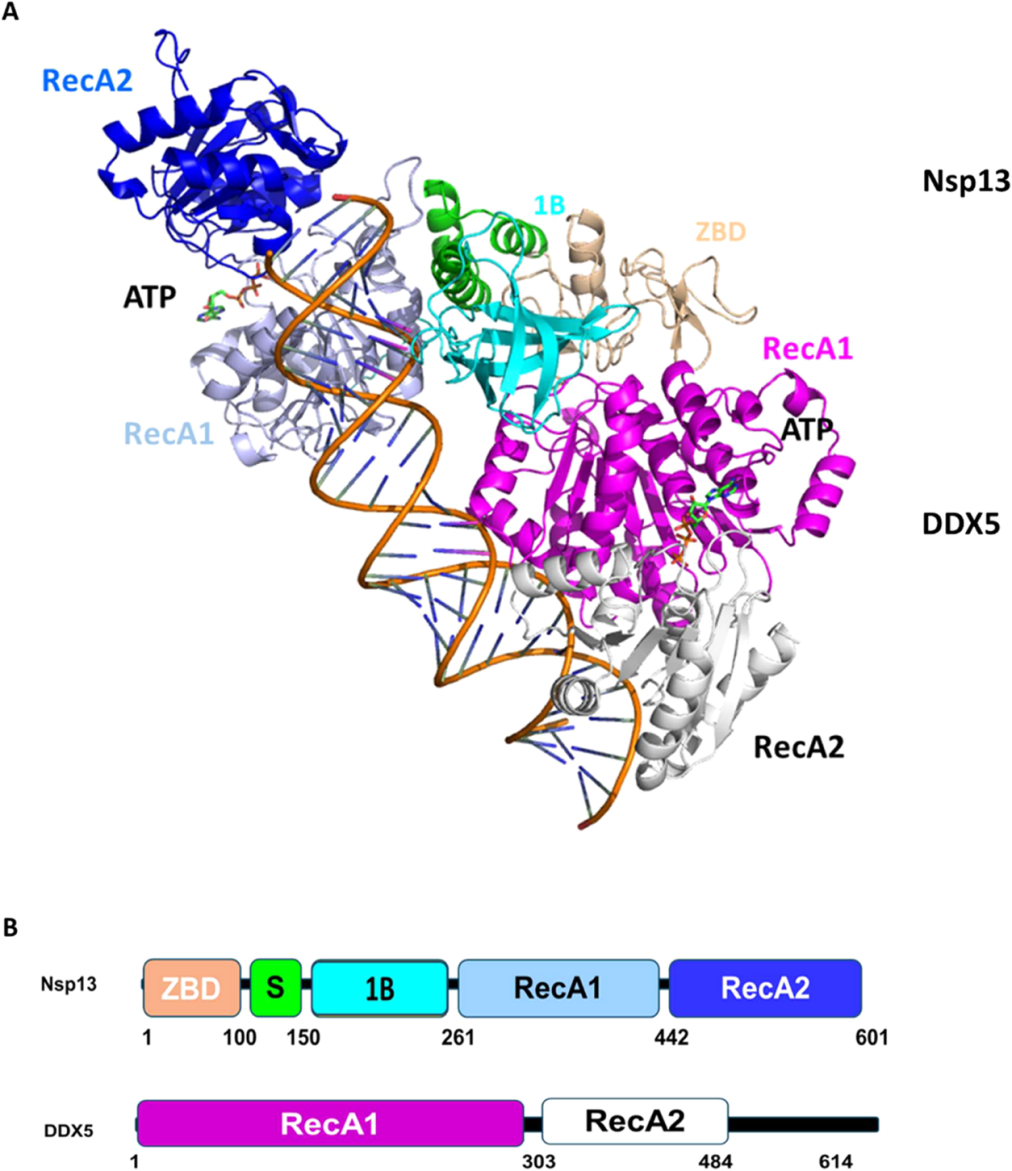
Model of the interaction between Nsp13 and DDX5, in complex with dsRNA. (A) Cartoon representation of the DDX5–Nsp13 complex computed using AlphaFold3.0. ATP molecules are drawn in stick. Main interactions are mediated by the RecA1 domain of DDX5 (prune) with the 1B (cyan) and the ZBD domain (salmon) of Nsp13. The dsRNA sequence is taken from the Nsp13_2_–RTC complex structure (pdb code 7rdz). (B) Color-coded domain organization of Nsp13 and DDX5.

## Methods

### Molecular Modeling

Molecular modeling of DDX5 in complex with ATP and in complex with Nsp13 and double-stranded RNA was conducted using artificial intelligence and employing AlphaFold3.0.^30^ The reliability of the predictions was assessed by both the local distance difference test (LDDT) score and a per-residue confidence score, with values higher than 90 meaning high confidence and below 50 low confidence.

### Recombinant Production of Nsp13 and DDX5

The optimized gene encoding the SARS-CoV-2 Nsp13, purchased from Eurofins Genomics Italy, was cloned into the pETM-13 expression vector (EMBL, Hamburg, Germany) to express a C-terminal His-tag protein. Two liters of LB/Kan was inoculated with an overnight preculture of BL21­(DE3) bacterial cells. The cells were previously grown at 37 °C and then induced at 0.7 OD600 by adding 0.2 mM IPTG and then incubated for 18 h at 18 °C. Bacterial pellet was resuspended in lysis buffer composed of 50 mM HEPES (pH 7.4), 500 mM NaCl, 5% (v/v) glycerol, and 2 mM DTT enriched with a cocktail of protease inhibitors (Roche), 10 μg/mL DNase and RNase. Then, a sonication step was applied to recover the soluble expressed protein after centrifugation. The soluble fraction was loaded onto a Ni-NTA resin (Qiagen) to carry out the first step of purification. Eluted Nsp13 was concentrated by using a centrifugal device (Amicon, Merck) and then loaded onto a Superdex 200 Increase 10/30 column (Cytiva) equilibrated with 50 mM HEPES (pH 7.4), 200 mM NaCl, 5% (v/v) glycerol, and 2 mM DTT. Purified Nsp13 was quantified and stored at −80 °C.

The gene encoding native full-length DDX5 was cloned into the pET30a vector to express an N-terminal His-tag protein. The expression was optimized using Rosetta 2 (DE3) cells, in particular a one-liter flask of LB/Kan/Chl was inoculated with 10 mL of an overnight preculture. The cells were grown at 37 °C, induced at 0.7 OD600 by adding 0.5 mM of IPTG, and then incubated for 18 h at 18 °C. The purification was carried out using a similar procedure to that adopted for Nsp13. Afterward, we also produced a C-terminal truncated form of DDX5 (1–484 amino acid region). The optimized DDX5 gene for *Escherichia coli* expression was synthesized and cloned into the pETM-13 expression vector (EMBL, Hamburg, Germany) to express a N-terminal His-tag protein. From this new gene, we also cloned and produced RecA1 (1–303) and RecA2 (303–484) domains of DDX5.

### CD Studies

All CD analyses were performed using a Jasco J-1500T spectropolarimeter equipped with a Peltier temperature control system (Model PTC-423-S). Molar ellipticity per mean residue, [θ] in deg cm^2^·dmol^–1^, was derived from the equation: [θ] = [θ]­obs·mrw·(10·*L*·C)^−1^, where [θ]­obs is the ellipticity measured in degrees, mrw is the mean residue molecular mass, *C* is the protein concentration in mg·mL^–1^, and *L* is the optical path length of the cell in cm. Far-UV spectra (between 195 and 260 nm) were recorded at 20 °C using a 0.1 cm optical path length cell and a protein concentration of 0.15–0.2 mg·mL^–1^. The samples were prepared by diluting the protein of interest in 20 mM sodium phosphate buffer (pH 7.4). Thermal denaturation experiments were performed by recording the CD signal at 222 nm between 0 and 80 °C.

### Biolayer Interferometry (BLI) Binding Studies

The freshly purified Nsp13 protein was covalently immobilized on an AR2G biosensor (Sartorius) using a standard protocol. Briefly, the sensor surface was first activated by injecting a mixture of 20 mM EDC and 10 mM Sulfo-NHS for 300 s, and then, the Nsp13 ligand (25 μg/mL in 10 mM NaAc, pH 5.5) was immobilized for 600 s. Finally, the activated sensor tips were blocked by injecting 1 M ethanolamine, pH 8.5, for 300 s. Then, the kinetics of association and dissociation to the immobilized ligand (Nsp13) were measured by diluting the analytes (DDX5, RecA1, and RecA2) in a range of 500–20 nM in 1× kinetic buffer (PBS 1×, 0.1% BSA, and 0.02 (v/v) % Tween 20). All kinetic assays on Octet R8 (Sartorius) were performed using 96-well black plates at 25 °C with an orbital shake speed of 1000 rpm. Octet Analysis Studio Software was used for data analysis.

### Microscale Thermophoresis (MST)

The equilibrium dissociation constant (*K*_D_) values were measured by using the Monolith NT.115 instrument (NanoTemper Technologies). The flDDX5 protein was fluorescently labeled according to the manufacturer’s protocol. A serial dilution of Nsp13 (20–0 μM range) was prepared in assay buffer (50 mmol/L Hepes, 200 mmol/L NaCl, 0.05% Tween 20, 5 mg/mL BSA, pH 7.5) and incubated with 5 nM purified labeled DDX5 protein for 15 min. For the displacement assay, 5 nm flDDX5 was incubated with 800 nM Nsp13 (a concentration that gives 80% saturation balancing between achieving a reasonable signal and a reasonable sensitivity). A serial dilution of unlabeled flDDX5 (3–0 μM), RecA1 (20–0 μM), and RecA2 (55–0 μM) was prepared in the assay buffer and incubated with the obtained DDX5–Nsp13 complex. The samples were loaded into Monolith NT.115 Standard Treated Capillaries, and experiments were carried out using 50% light-emitting diode power and 50% MST. The experimental points were interpreted using Hill sigmoidal fitting from triplicate reads of measurements.

### Non-Denaturing Gel-Based Helicase Assay

The helicase activity of Nsp13, DDX5, RecA1, and RecA2 domains was monitored by measuring the conversion of 50 nM dsRNA 18/38 mer FAM into a ssRNA 18 mer FAM. Reactions were performed in a helicase buffer (20 mM Tris–HCl pH 8, 2 mM DTT, 70 mM KCl, 2 mM MgCl_2_, 6U RNasin) and started with the addition of 4 mM ATP. After 20 min of incubation at 30 °C, reactions were stopped by adding 6x gel loading buffer (Thermo Fisher Scientific, Waltham, MA) and run on 10% TBE-polyacrylamide 19:1 + 0.1% SDS gel at 40 V for about 3 h in a 1X TBE + 0.1% SDS buffer at 4 °C in a mini-PROTEAN electrophoresis system (Bio-Rad, Hercules, CA). Substrates and products were quantified by laser scanning densitometry with a Typhoon-TRIO (GE Healthcare, Uppsala, Sweden). When Nsp13 was used in the presence of DDX5 and RecA domains, proteins were preincubated for 10 min at RT before the addition of dsRNA and ATP. RNA oligonucleotides were purchased from Biomers.net GmbH (Ulm, Germany). The sequences of the substrates used are

ssRNA 38 mer: 5′- AUGAAGGUUUGAGUUGAGUGGAGAUAGUGGAGGGUAGU-3′

ssRNA 18 mer FAM: 3′- UACUUCCAAACUCAACUC-5′ FAM

For dsRNA, oligonucleotides were mixed at a 1:1 (M/M) ratio in annealing buffer (30 mM HEPES-KOH, pH 7.4, 100 mM KCl, 2 mM MgCl_2_, 50 mM NH_4_Ac) at a final concentration of 500 nM, heated at 95 °C for 5 min, and then slowly cooled at room temperature.

### Quantification and Statistical Analysis

The intensity of the bands was measured by ImageJ densitometry, and the values were used to calculate the percentage of the helicase activity. GraphPad Prism 9.0 software was used to draw the graphic bars.

## Supplementary Material



## Notes

Cartoon representation of the DDX5 structure, computed using Alphafold3.0 (Figure S1); overlay of BLI sensorgrams obtained for the binding of different concentrations of DDX5ΔC, RecA1, and RecA2 domains of DDX5 to the immobilized Nsp13 (Figure S2); direct binding of Nsp13 to fluorescent RecA1 by MST (Figure S3); and cartoon representation of Nsp13 RecA domains in Nsp13–DDX5 complex (Figure S4) (PDF)
