# Supplementary material for: Human Helicase DDX5 is Hijacked by SARS-CoV‑2 Nsp13 Helicase to Enhance RNA Unwinding
Source: ACS Omega. 2025 Jul 31;10(31):34941–50. doi: 10.1021/acsomega.5c04271 (PMC12355267; doi:10.1021/acsomega.5c04271)
Supplement: Supplementary file 1 [file ao5c04271_si_001.pdf]

The human helicase DDX5 is hijacked by SARS-CoV-2 Nsp13 helicase to enhance RNA unwinding

Giovanni Barra[a±], Alessia Ruggiero[a±], Valeria Napolitano[a], Camilla Lodola[b], Massimiliano Secchi[b], Maria Michela Pallotta[b], Viviana Benincasa[b], Francesco Leone[b], Giovanni Maga[b]*, Rita Berisio[a]*

[a]*Institute of Biostructures and Bioimaging, C.N.R., Napoli, Italy*

[b]*Institute of Molecular Genetics, C.N.R., Pavia, Italy*

Correspondence to rita.berisio@cnr.it; giovanni.maga@igm.cnr.it

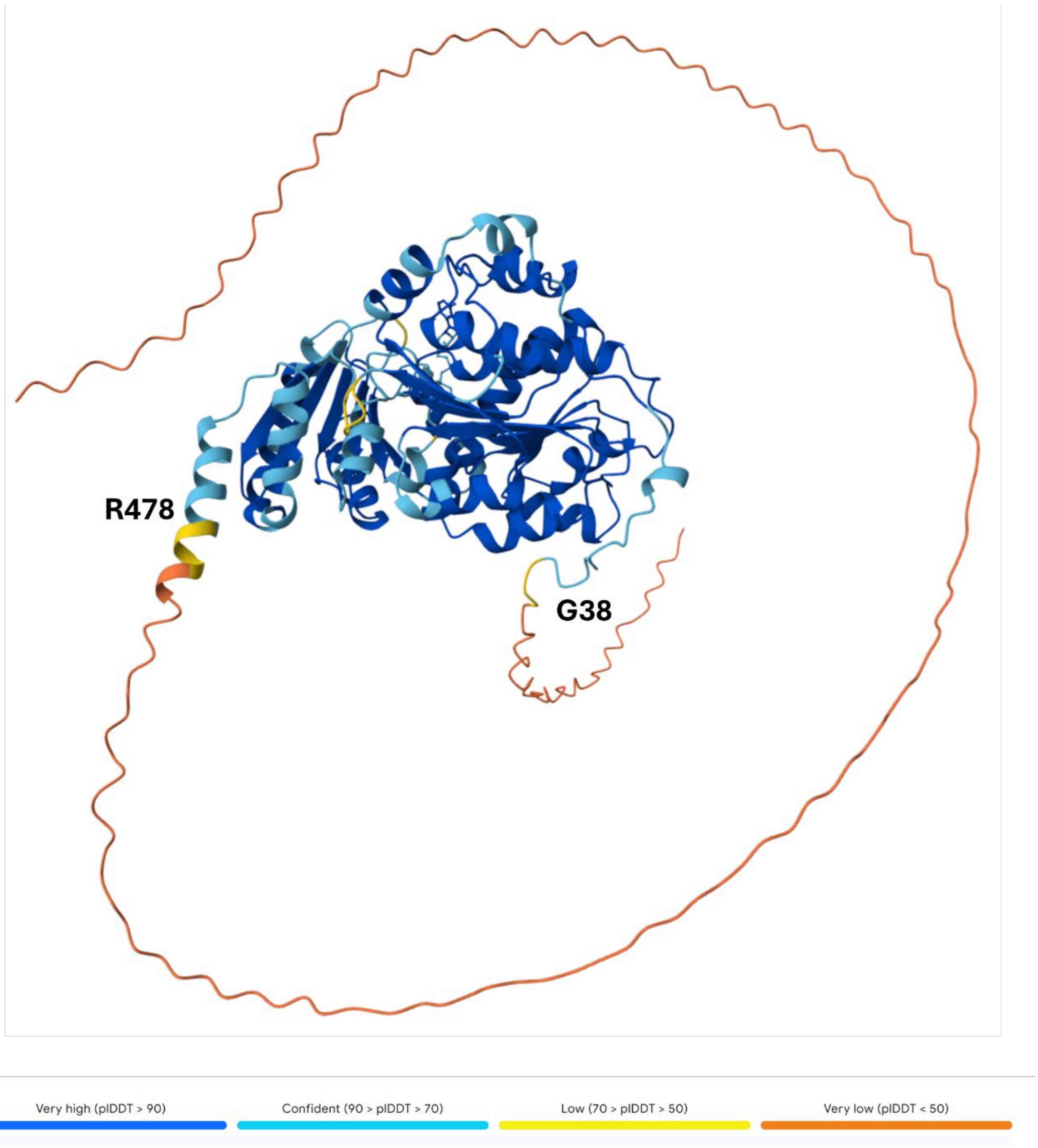

Figure S1. Cartoon representation of the DDX5 structure, computed using Alphafold3.0. The color code is reported in the bottom panel. Regions M1-G38 and 479-614 show low confidence (plDDT <50). The remaining structure shows plDDT >70, apart from the loops T435-T435 and A304-A309 (70>plDDT>50)

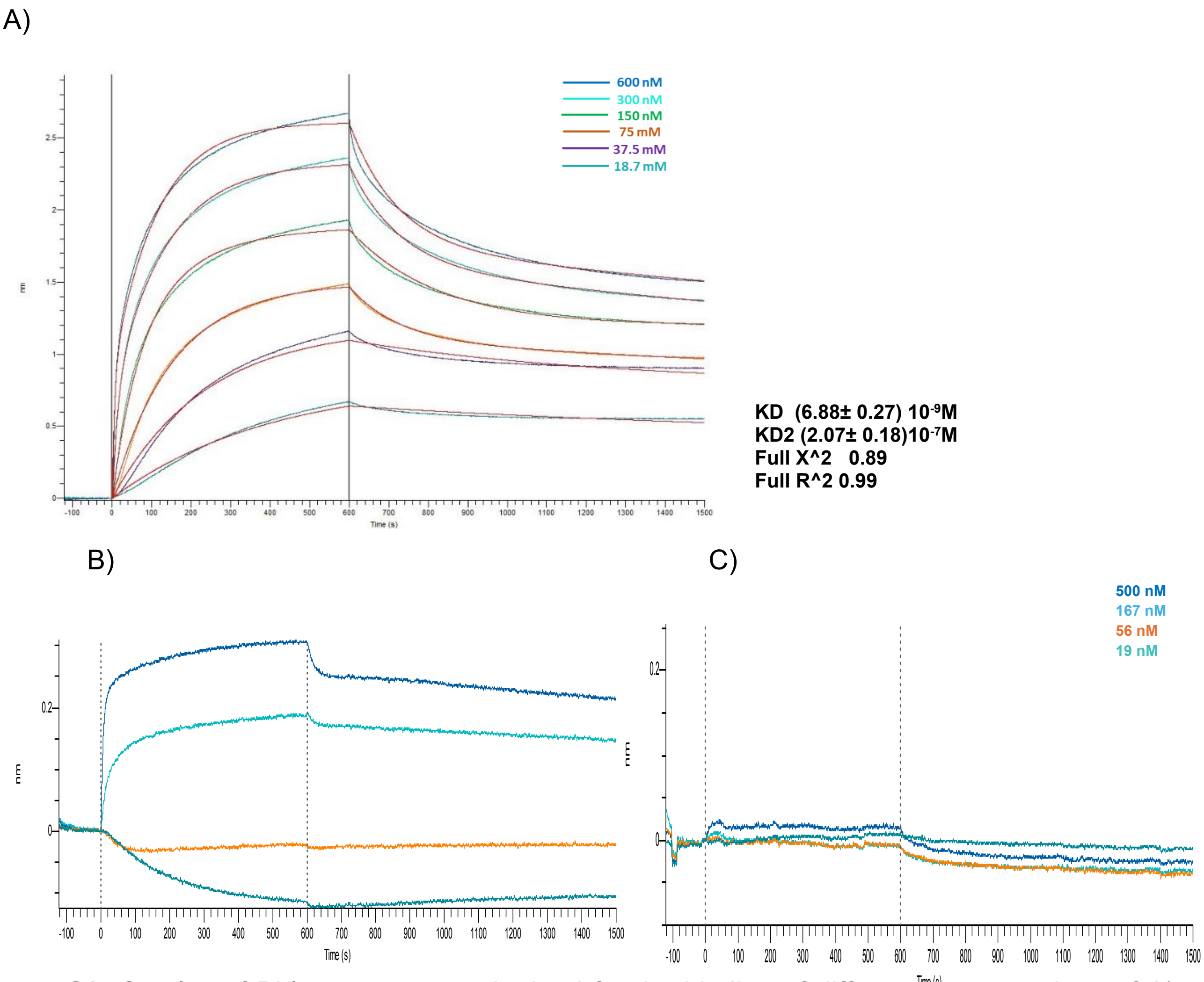

Figure S2. Overlay of BLI sensorgrams obtained for the binding of different concentrations of A) DDX5ΔC B) RecA1 domain and C) RecA2 domain to the immobilized Nsp13. Binding constants (KD/KD2 values) could be obtained only for DDX5ΔC.

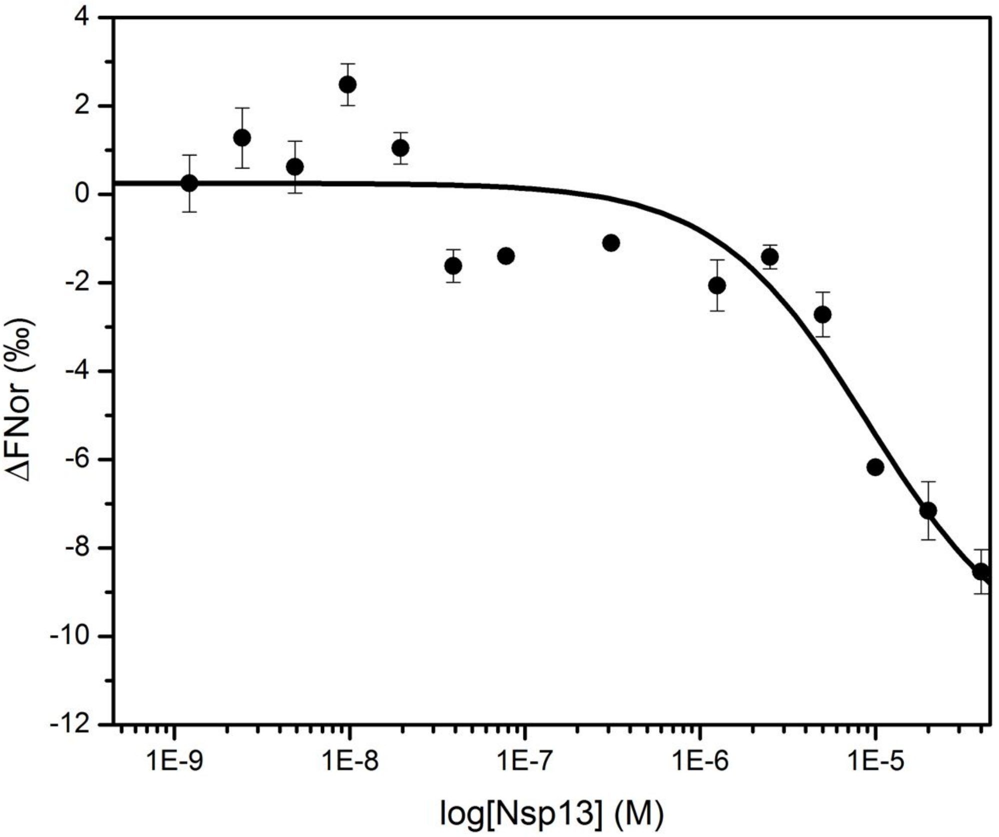

Figure S3. Direct binding of Nsp13 to fluorescent RecA1 by MST. The error bars represent the SD of each data point calculated from three independent thermophoresis measurements.

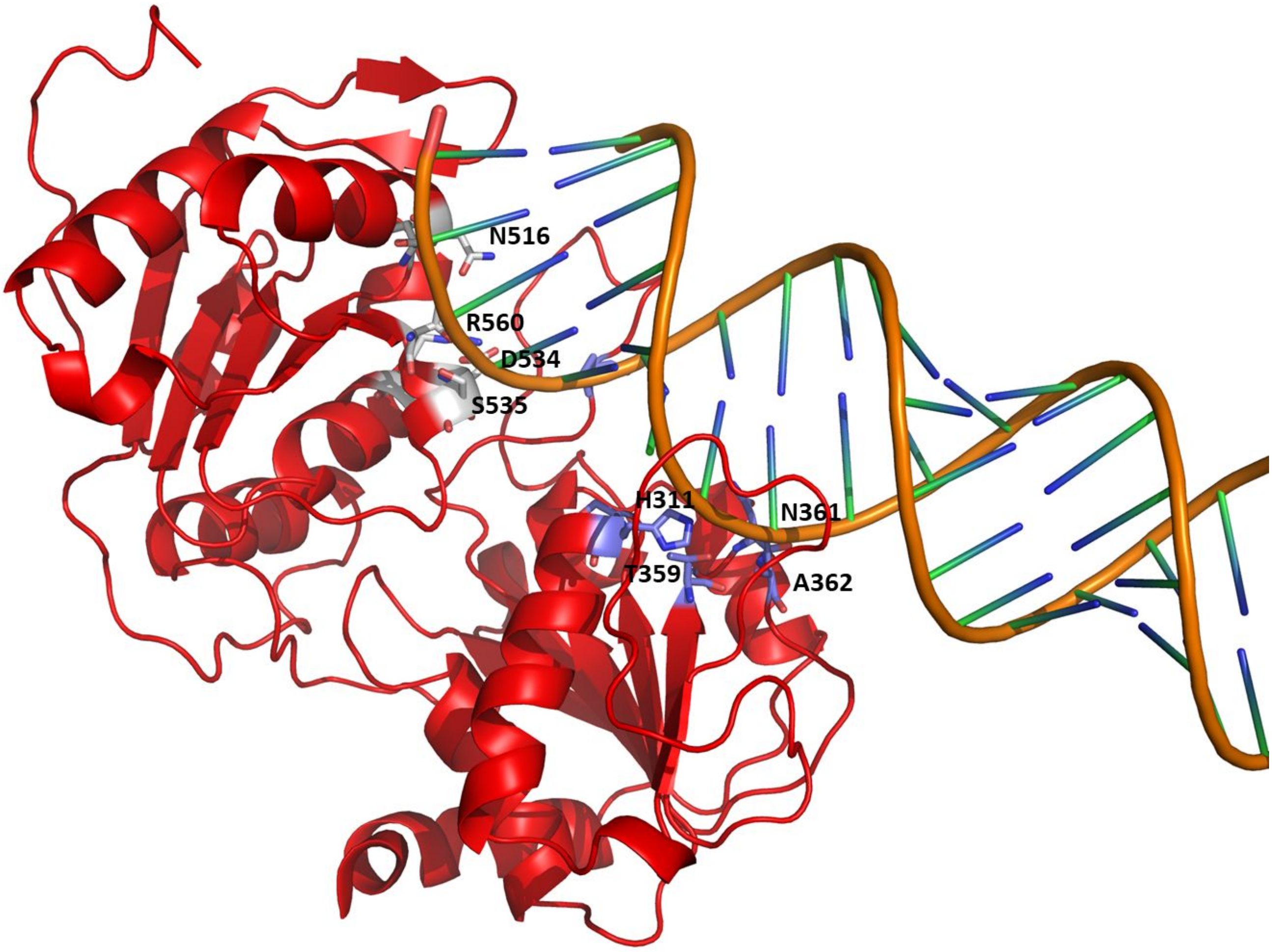

Figure S4. Cartoon representation of Nsp13 RecA domains in Nsp13-DDX5 complex. Residues involved in RNA binding are drawn in stick representation.
